# Neuron-Type Specific Functions of DNT1, DNT2 and Spz at the Drosophila Neuromuscular Junction

**DOI:** 10.1371/journal.pone.0075902

**Published:** 2013-10-04

**Authors:** Ben Sutcliffe, Manuel G. Forero, Bangfu Zhu, Iain M. Robinson, Alicia Hidalgo

**Affiliations:** 1 School of Biosciences, University of Birmingham, Birmingham, United Kingdom; 2 Peninsula College of Medicine and Dentistry, University of Plymouth, Plymouth, United Kingdom; University of Florida, United States of America

## Abstract

Retrograde growth factors regulating synaptic plasticity at the neuromuscular junction (NMJ) in Drosophila have long been predicted but their discovery has been scarce. In vertebrates, such retrograde factors produced by the muscle include GDNF and the neurotrophins (NT: NGF, BDNF, NT3 and NT4). NT superfamily members have been identified throughout the invertebrates, but so far no functional *in vivo* analysis has been carried out at the NMJ in invertebrates. The NT family of proteins in Drosophila is formed of DNT1, DNT2 and Spätzle (Spz), with sequence, structural and functional conservation relative to mammalian NTs. Here, we investigate the functions of Drosophila NTs (DNTs) at the larval NMJ. All three DNTs are expressed in larval body wall muscles, targets for motor-neurons. Over-expression of DNTs in neurons, or the activated form of the Spz receptor, *Toll* ^*10b*^, in neurons only, rescued the semi-lethality of *spz* ^2^ and *DNT1* ^*41*^ *, DNT2* ^*e03444*^ double mutants, indicating retrograde functions in neurons. In *spz* ^2^ mutants, *DNT1* ^*41*^ *, DNT2* ^*e03444*^ double mutants, and upon over-expression of the DNTs, NMJ size and bouton number increased. Boutons were morphologically abnormal. Mutations in *spz* and *DNT1,DNT2* resulted in decreased number of active zones per bouton and decreased active zone density per terminal. Alterations in DNT function induced ghost boutons and synaptic debris. Evoked junction potentials were normal in *spz* ^2^ mutants and *DNT1* ^*41*^ *, DNT2* ^*e03444*^ double mutants, but frequency and amplitude of spontaneous events were reduced in *spz* ^2^ mutants suggesting defective neurotransmission. Our data indicate that DNTs are produced in muscle and are required in neurons for synaptogenesis. Most likely alterations in DNT function and synapse formation induce NMJ plasticity leading to homeostatic adjustments that increase terminal size restoring overall synaptic transmission. Data suggest that Spz functions with neuron-type specificity at the muscle 4 NMJ, and DNT1 and DNT2 function together at the muscles 6,7 NMJ.

## Introduction

The Drosophila larval neuromuscular junction (NMJ) is a standard and successful paradigm used to probe questions into synapse formation, function, synaptic plasticity (i.e. potentiation, depression) and structural plasticity (e.g. of bouton number, terminal length, dendritic arbors) [1]. It is a predominantly glutamatergic synapse, similar to most mammalian central synapses. It has long been thought that retrograde factors produced by the muscle regulate pre-synaptic events and synaptic efficacy [2], but identified factors are scarce. The only retrograde factor known in flies is the muscle derived TGF ligand Glass bottom boat (Gbb), which functions in motor-neurons via the receptor Wishful-thinking and the MAD pathway [3]. Anterograde factors known in Drosophila are Jelly Belly (Jeb), the ligand for Anaplastic lymphoma kinase (Alk) and Wingless (Wg), ligand of Frizzled in the muscle, both secreted presynaptically to promote synapse formation and stability [4,5]. At the mammalian NMJ synapse, neurotrophins (NTs) – NGF, BDNF, NT3 and NT4 - are secreted by both muscle and motor-neurons, and function both retrogradely and anterogradely to influence connectivity, synapse formation, synaptic transmission and potentiation [6]. The lack of NT studies at invertebrate synapses represents a notable gap for the general understanding of synaptic structure and function across the animals.

The NT superfamily members in Drosophila are encoded by the genes *DNT1* (*spz2*), *DNT2* (*spz5*) and *spätzle* (*Spz*) [7,8,9,10,11]. Using bioinformatics, homologues of both the mammalian NTs and Drosophila *spz* have been identified in other invertebrates [12], implying a shared ancestral gene followed by gene duplications. In Drosophila, Spz was characterised using biochemical, structural and bioinformatic analyses as a secreted protein containing an NGF motif [7,9,13] and DNT1 and DNT2 were identified by sequence and structural conservation relative to vertebrate BDNF [11]. Using bioinformatics tools, DNT1 and DNT2 appear to be more closely related to the mammalian NTs than Spz [11], although so far only the crystal structure of Spz is known. Spz, DNT1 and DNT2 have the same protein structure as that of all other known NT superfamily members. They are characterised by a signal peptide, followed by an unstructured pro-domain, and a conserved cystine-knot domain characteristic of the NT super-family and distinct from other cystine-knots, e.g. of the PDGF and TGFinsertSymbolb protein families [7,9,10,11,13,14,15]. The characteristic spacing of Cysteines and key amino acids that form the cystine-knot are conserved throughout the NTs in vertebrates, all identified invertebrate NTs, and DNT1, DNT2 and Spz. DNT1 also has a disordered carboxyl terminal extension [10,11], reminiscent of that found in Amphioxus NT [16]. The 3D structure of the crystallised Spz cystine-knot domain can be superimposed with that of NGF [14,17,18]. As with mammalian NTs, DNTs are cleaved to release the mature cystine-knot. Contrary to other related cystine-knot proteins, such as Coagulogen, which is monomeric, the DNTs dimerise for function, like mammalian NTs do. The active form of the DNTs is the cleaved, mature cystine-knot form and only cleaved Spz or DNT1 can trigger signalling and rescue their loss of function in mutants *in vivo* [11,19,20,21,22].

There is also functional conservation amongst the DNTs and vertebrate NTs. *DNT1, DNT2* and *spz* are expressed in embryonic muscles (target of motor-neurons), and other target tissues, such as the midline (target of interneurons) and the optic lobe (target of photoreceptor neurons) [11]. Mutations in *DNT1*, *DNT2* or *spz* result in increased apoptosis in the central nervous system (CNS), and targeting errors at the embryonic NMJ [11]. Conversely, over-expression of cleaved *DNT1CK, DNT2CK* or *spzCK* rescues neuronal survival, and over-expression of *DNT1* at the muscle also interferes with targeting [11]. Thus, DNT1, DNT2 and Spz promote neuronal survival and connectivity. At least DNT1 functions in a target-dependent manner, since RNAi knock-down restricted to the midline induces apoptosis in the CNS [11]. Conversely, *DNT1CK* over-expression at the midline can rescue neuronal survival in the CNS [11]. Remarkably, in the embryonic musculature *DNT1*, *DNT2* and *spz* are expressed in overlapping and complementary patterns and are required by different neuronal types. Most intriguingly, mutations in *spz* affect targeting by the SNa motoraxons, whereas *DNT1,DNT2* double mutants have defects in targeting by the ISNb/d motoraxons [11]. This strongly suggests that the DNTs are required differentially by distinct motor-neurons.

The receptor for Spz is Toll [15], but the receptors for DNT1 and DNT2 have not yet been identified. The structural and biochemical properties of Spz binding to Toll have been extensively characterised in comparison to binding of NGF to its p75^NTR^ and Trk receptors [8,13,15,23]. Expression of *Toll* at the NMJ has been reported post-synaptically in muscles but not pre-synaptically in neurons [24,25,26,27]. In the embryo, Toll is localised in embryonic CNS axons [11], and it functions in muscle to inhibit synaptogenesis [25,26,27,28] and in neurons to promote cell survival [11]. Whether the Drosophila NT protein family is required at the synapse is unknown.

Here, we show that *Drosophila* NTs, are expressed in the larval body wall muscle, and are required in neurons at the NMJ for synaptogenesis with neuron-type specificity.

## Materials and Methods

### Genetics

The wild-type control was *yw*. Details of mutants and UAS over-expression lines are provided in [11,13]. For UASspzCysknot (UASspzCK) we used UASactivated-spz (line 14, gift of Jean Marc Reihhart) [20] and UASspzΔN (gift of Tony Ip) [19]; UASDNT1CK3’+ includes the Carboxyl terminal extension after the cystine-knot [11]. Other genotypes were generated by conventional genetics. For detailed genotypes, see Table S1.

### Immunohistochemistry, *in situ* hybridisations and microscopy

*In situ* hybridisations with RNA probes, immunohystochemistry and image capture were carried out following standard procedures. Primary antibodies were used at the following dilutions: anti-Brp/nc82 at 1:100 (Iowa Hybridoma Bank); anti-Dlg at 1:20 (Iowa Hybridoma Bank); anti-HRP at 1:250 (Jackson ImmunoResearch). Secondary antibodies were anti-Rabbit and anti-Mouse directly conjugated to Alexa 488 or Alexa 660 at 1:250 (Molecular Probes).

### Bouton counts, and axonal terminal and active zone density measurements

Boutons were labelled with anti-Dlg, identified as described in [29] and counted manually. Only abdominal segment A4 was analysed. Axonal terminal length was measured by tracing HRP+ axons with the mouse using freehand line tracing and the region of interest (ROI) Manager in ImageJ. To quantify active zone density, we purposely developed an automatic ImageJ plugin called DeadEasy Synapse that measures the volume occupied by nc82 (also known as Bruchpilot or Brp) voxels, and which will become freely available after publication. In each confocal stack of images corresponding to one NMJ in one specimen, an ROI was selected around the axonal NMJ terminal to exclude background. Multiple confocal stacks were then batch processed using the DeadEasy Synapse ImageJ plugin, which provides total voxel occupancy by Brp/nc82 signal at each terminal. Data using this plug-in are provided as “DeadEasy Synapse”: mean number of voxels occupied by Brp/nc82. Brp/nc82 dots were also counted manually: each confocal stack was opened in ImageJ and each active zone labelled with anti-nc82 was carefully determined, marked and counted using the ImageJ Cell Counter plug-in. These data are provided as number of Brp/nc82 dots or active zones.

### Electrophysiology

Wandering third instar larvae were dissected in HL3 [30] containing either 0.7 mM or 1.8 mM CaCl_2_. Nerves were cut near the ventral ganglion and stimulated using a suction electrode and isolated pulse stimulator Digitimer DS2A (constant current modification), with a current double that needed to initiate a compound response. All recordings were made intracellularly at ambient room temperature (~22 °C) using microelectrodes filled with 3 M KCl, in A2 or A3, for *DNT1* ^*55*^ *, DNT2* ^*e03444*^ in muscle 6 or 7 and for *spz* ^2^ in muscle 4. A2 or A3 abdominal segments were used because the motor-neurons innervating these segments are shorter and easier to find and access. In 1.8 mM extracellular Ca^2+^ muscle 6 resting membrane potentials were -63.1 ± 1.2 mV (n = 13) and -63.0 ± 0.8 mV (n = 19, *p* >0.05) and muscle resistances were 9.4 ± 0.5 MInsertSymbolΩ (n = 14) and 9.1 ± 0.9 MInsertSymbolΩ (n = 8) for yw and *DNT1* ^*55*^ *, DNT2* ^*e03444*^ larvae, respectively. In 0.7 mM extracellular Ca^2+^ muscle 6 resting membrane potentials were -64.3 ± 0.9 mV (n = 9) and -61.8 ± 1.7 mV (n = 10, *p* >0.05) and muscle resistances were 9.9 ± 0.9 MInsertSymbolΩ (n = 14) and 10.0 ± 0.5 MInsertSymbolΩ (n = 10) for yw and *DNT1* ^*55*^ *, DNT2* ^*e03444*^ larvae, respectively. In 1.8 mM extracellular Ca2+ muscle 4 resting membrane potentials were -51.4 ±2.3 mV (n =9) and -51.6 ±1.7 mV (n =12, p >0.05) and muscle resistances were 10.1 ±0.8 MInsertSymbolΩ (n =9) and 9.2 ± 0.3 MInsertSymbol Ω for *yw* and *spz* ^2^ larvae, respectively. In 0.7 mM extracellular Ca2+ muscle 4 resting membrane potentials were -55.1 ± 1.6 mV (n =8) and -51.8 ± 1.8 mV (n =8, p >0.05) and muscle resistances were 8.9 ± 0.6 MInsertSymbolΩ (n =8) and 9.4 ± 0.8 MInsertSymbolΩ for *yw* and *spz* ^2^ larvae, respectively. Data, filtered at 1 kHz and digitized at 10 kHz, were acquired using an Axopatch 200B amplifier and a Digidata 1320A data acquisition board (Molecular Devices Data) using pClamp8.02 (Molecular Devices) and analysed with Clampfit8.02 (Molecular Devices) or MiniAnalysis (Synaptosoft). EJP amplitude histograms were constructed by averaging 50 separate events from an individual muscle cell, stimulated at 1 Hz, and then calculating the mean response from at least 8 larvae per line. Spontaneous release events were recorded for 600 sec from muscle 4 and 120 sec from muscle 6 without any electrical stimulation; all events were analysed and mean mEJP amplitude from individual larvae were averaged to generate the histograms.

### Statistical analysis

Statistical analysis was carried out using SPSS, and the null hypothesis in all cases was that there is no phenotypic difference between wild-type controls and other genotypes. All samples within a group were first analysed together, and subsequently comparisons to controls were carried out applying multiple comparison corrections. Categorical data (e.g. survival assays and proportions of NMJs with ghost boutons or debris) were analysed using Chi-square, and Bonferroni corrections for comparisons to controls. For continuous data (e.g. number of boutons, active zones, and electrophysiology data), kurtosis and skewness were used to test if the distributions were normal and Levene’s test for homogeneity of variance. All samples were either distributed normally or assumed to be distributed normally if the majority were. One Way ANOVA was carried out to compare whole sets of samples, and post-hoc Dunnett tests were carried out for comparisons of individual samples to controls. Significance was set at p<0.05. For details on statistical analyses, see Table S2.

## Results

### *spz* and DNTs are expressed in muscles and are required in neurons

**Figure 1:**
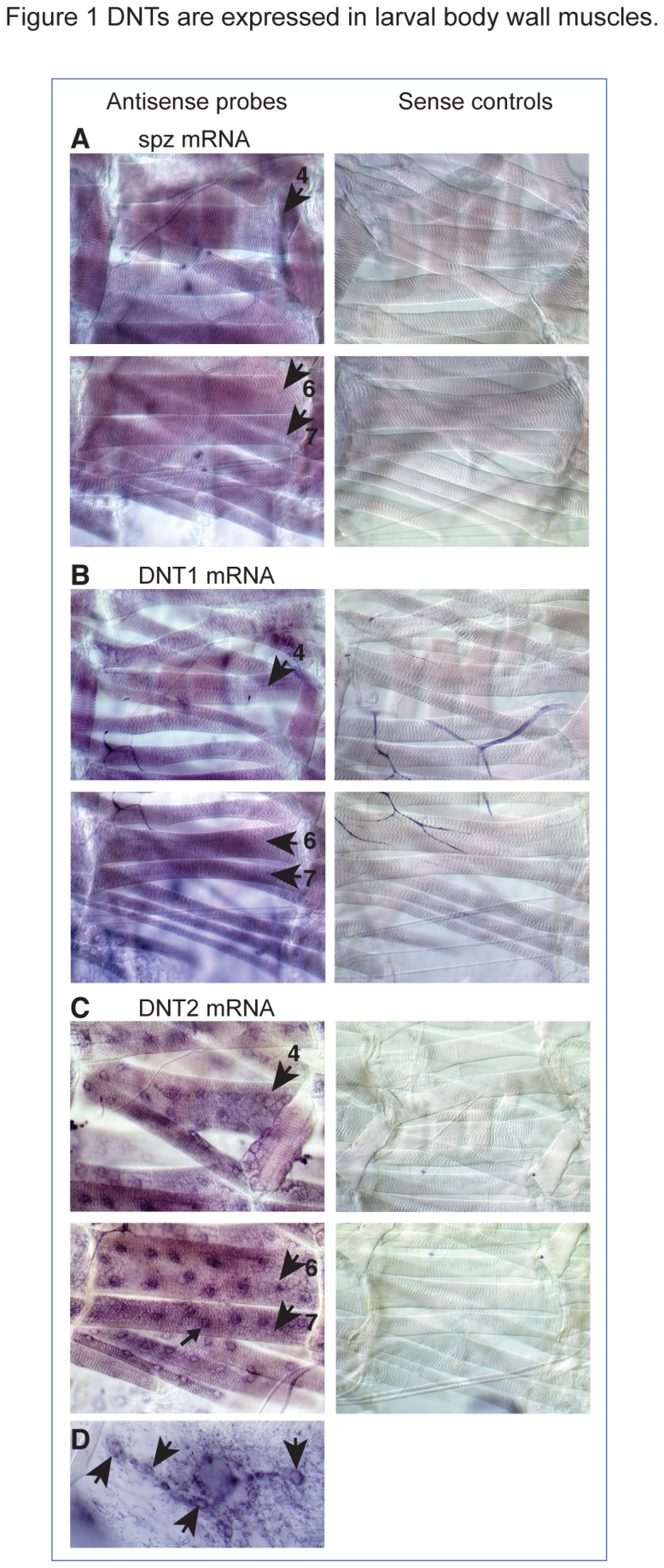
DNTs are expressed in larval body wall muscles. *In* *situ* hybridisations showing the distribution of *DNT* transcripts in third instar larval fillet preparations, sense control probes on the right. (A-C) *spz, DNT1* and *DNT2* transcripts are distributed uniformly in all muscles, including muscles 4, 6, 7, 12, 13 (arrows). (C) *DNT2* transcripts are present around muscle nuclei (small arrow), and (D) at synaptic boutons (arrows).

Transcripts for *spz, DNT1* and *DNT2* were found distributed uniformly throughout the larval body-wall musculature (Figure 1A-C). *DNT2* transcripts were also found surrounding the muscle nuclei (Figure 1C,D), and at synaptic boutons (Figure 1D). We did not find synaptic localisation of *DNT1* or *spz* transcripts.

**Figure 2:**
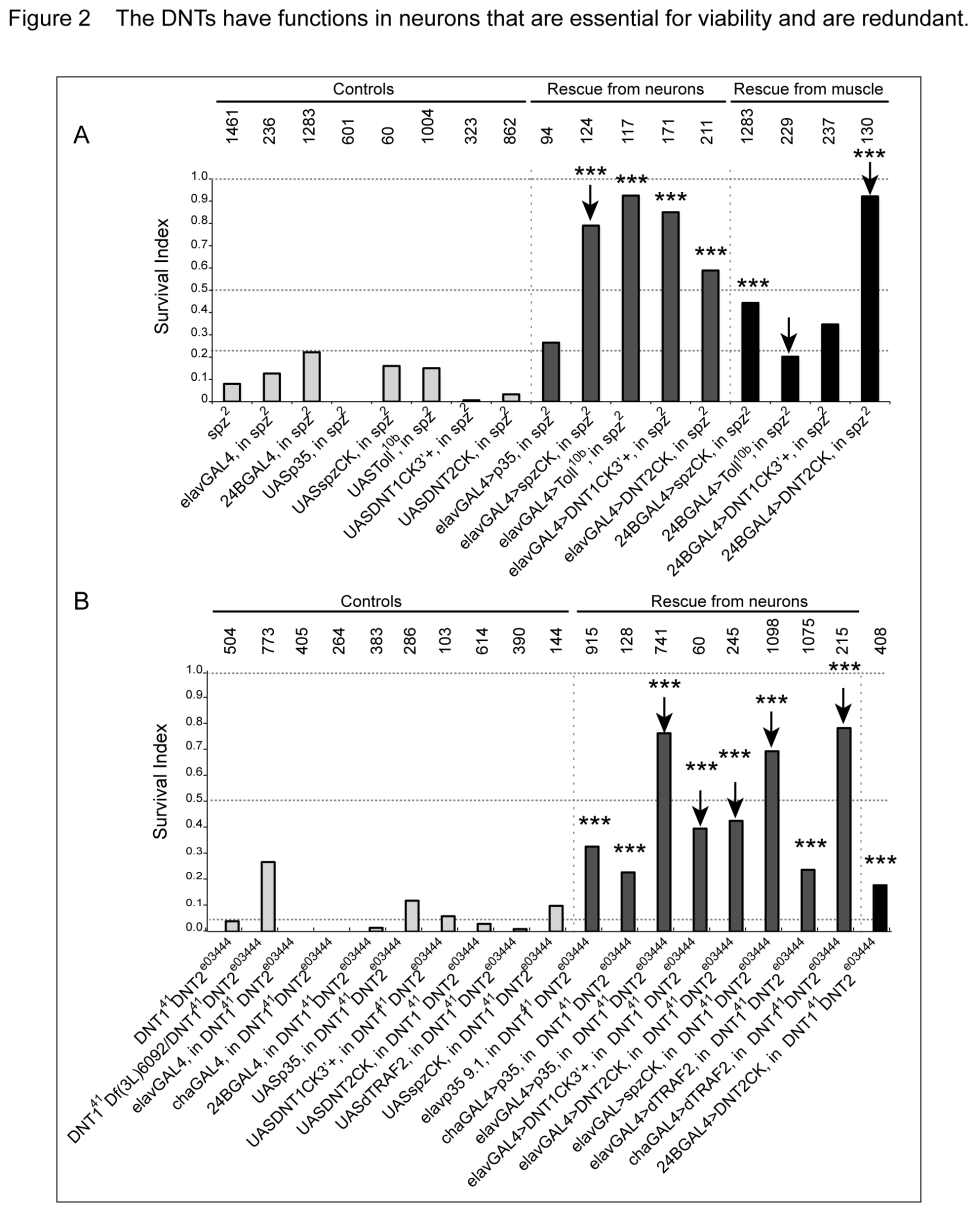
The DNTs have neuronal functions that are essential for viability and are redundant. Survival index is the ratio of homozygous to heterozygous pupae, relative to the Mendelian expectation for wild-type viability, given by S.I. = 2x No. Homozygous Tb^+^ /No.TM6B Tb^-^. For wild-type, S.I.=1. *elavGAL4* drives expression in all neurons and *24BGAL4* in all muscles. (A) Rescue of *spz* ^2^ semi-lethality expressing *p35, activated* *spzCK, DNT1CK3*’*+, DNT2CK* or *Toll* ^*10b*^ in neurons. Note that expression of activated *spzCK* (arrow) and *Toll* ^*10b*^ in neurons rescues the *spz* ^2^ lethality, expression of *DNT2CK* in muscle also rescues (arrow), but expression of *Toll* ^*10b*^ in muscle does not (arrow). (B) Rescue of *DNT1* ^*55*^ *DNT2* ^*e03444*^ double mutant semi-lethality at 18°C by *p35, DNT1CK3*’*+, DNT2CK, spzCK* and *dTRAF2* over-expression in neurons. Note that expression of only *DNT1CK3*’*+* or *DNT2CK* in neurons partially rescue the semi-lethality of the double mutant (arrow), indicating that they have distinct functions. Activated *spzCK* can also rescue the lethality of the double mutants, indicating redundant functions (arrow). *Elav-p35* is a fusion of the elav promoter upstream of p35, independently of GAL4. GAL4 drivers: *w;elavGAL4* (all neurons), *w;chaGAL4* (cholinergic neurons) or *w; 24BGAL4* (muscles). Numbers above bars indicate sample sizes n=number of pupae scored. Statistical tests: Chi-square with Bonferroni correction, *p<0.05, **p<0.01, ***p<0.001. For detailed genotypes see Table S1 and for statistical details, see Table S2.

To ask whether muscle expression might reflect a functional requirement in neurons or in muscle, we exploited the semi-lethality of the mutants and tested whether this could be rescued by over-expression in neurons or muscle. The *spz* ^2^ mutant allele is a point mutation in the pro-domain of Spz that is thought to interfere with its secretion [13]. *spz* ^2^ mutant flies are homozygous semi-lethal, with 7% adult escapers (Figure 2A). Thus, we first tested whether expressing activated *spz* (*spzCK, also known as C106*) or activated *Toll* (*Toll* ^*10b*^) in all neurons (*elavGAL4*) or in muscles (*24BGAL4*) could rescue the semi-lethality of homozygous *spz* ^2^ mutants. Over-expression of the baculovirus apoptosis inhibitor *p35* in neurons in *spz* ^2^ mutants resulted in a two-fold increase in fly survival (Figure 2A), consistent with the neurotrophic functions of the DNTs. Over-expression of *spzCK* or *Toll* ^*10b*^ in neurons of *spz* ^2^ mutants rescued fly survival to wild-type levels (Figure 2A). This indicates that the lethality of *spz* ^2^ mutants is due to abnormal Spz function in the CNS. Expression of *spzCK* in muscles also rescued *spz* ^2^ viability (Figure 2A). Since Spz is a secreted protein, it may conceivably have retrograde, as well as anterograde or autocrine functions, although so far there is no evidence that over-expressed *spzCK* is secreted. Importantly, expression of *activated*-*Toll* ^10b^ in muscles failed to rescue the *spz* ^2^ semi-lethality (Figure 2A). Since the semi-lethality of *spz* ^2^ mutants can be rescued by activated Toll expression in neurons only, these data suggest that Spz has retrograde functions from muscle to neurons and that this is essential for fly viability.

We have previously shown that the DNTs are structurally related and have redundant functions in other contexts [11]. Thus, we tested whether over-expression of *activated DNTs* in neurons could rescue the semi-lethality of *spz* ^2^ mutants. Expression of either activated *DNT1CK3*’*+* or activated *DNT2CK* in all neurons rescued the semi-lethality of *spz* ^2^ mutants (Figure 2A). This suggests that DNT1 and DNT2 may activate other Toll-like receptors and converge in the same downstream signalling pathway as Toll. Over-expression of *DNT2CK* in muscles was equally effective (Figure 2A). Altogether, these data show that both DNT1 and DNT2 can substitute for Spz, and suggest that at least DNT2 may also function retrogradely from the muscle.

In a similar fashion, we exploited the semi-lethality of *DNT1 DNT2* double mutants to test whether the DNTs are required in neurons. *DNT1* ^*41*^ *, DNT2* ^*e03444*^ and *DNT1* ^*41*^ *, Df*(*3L*)*6092/ DNT1* ^*41*^ *, DNT2* ^*e03444*^ double mutants are semi-lethal when reared at 18°C as progeny of a stock balanced over TM6B (Figure 2B). This temperature sensitive semi-lethality was rescued by blocking apoptosis in all neurons by expressing *p35* under the direct control of the *elav* promoter, or by over-expressing *p35* with *chaGAL4* or *elavGAL4* - consistent with the neurotrophic functions of the DNTs (Figure 2B). Rescue of double mutant semi-lethality was also seen with by the over-expression of *DNT1CK, DNT2CK* or *spzCK* in neurons (Figure 2B). Interestingly, this semi-lethality was also rescued when the NFInsertSymbolkB pathway was activated by the over-expression of *dTRAF2* in cholinergic neurons (*chaGAL4*). The fact that rescue with p35 and dTRAF2 have a stronger effect than with either DNT1 or DNT2 can be explained by the fact that the double mutant lacks two factors and only one of them is over-expressed, whereas p35 and dTRAF2 affect all neurons and signalling mechanisms presumably shared by both DNT1 and DNT2. Since Toll is known to activate dTRAF2 and NFInsertSymbolkB signalling, this suggests that Spz, DNT1 and DNT2 share common downstream signaling pathways.

To conclude, the expression profile and the genetic tests show that DNTs are expressed in muscle, and have non-autonomous and potentially retrograde functions in neurons.

### Altered DNT function affects bouton number and terminal size with neuron type specificity

We then asked whether morphological aspects of the NMJ were affected by altered DNT function. Synaptic boutons were visualised by the co-localisation of pre-synaptic anti-HRP and the PSD-95 homologue anti-Discs Large (Dlg), which is predominantly post-synaptic, and were counted manually and also normalised over muscle surface area (MSA) [31](Table 1). Axonal terminals were visualised with anti-HRP and the additive length of all axonal branches per NMJ was measured using ImageJ and normalised to MSA. Muscles 6,7 MSA was reduced in *DNT1* ^*41*^ *, DNT2* ^*e03444*^ double mutant larvae compared to wild-type (Table 1, p<0.001), and muscle 4 MSA was larger upon neuronal over-expression of *spzCK* or *DNT1CK3*’*+* (Table 1, p<0.001 and p<0.05 respectively).

**Table 1:** Raw data: quantification of NMJ phenotypes.

	**Muscle 4 NMJ**						
**Genotype**	**MSA µm^2^10^-3^Mean (±s.e.m.)**	**No. of Boutons Mean (±s.e.m)**	**Terminal length µm Mean (±s.e.m.)**	**DeadEasy Synapse Voxel/ Bouton. Mean (±s.e.m.)**	**Active zone count/ Bouton. Mean (±s.e.m.)**	**DeadEasy Synapse Voxel/ Terminal length. Mean (±s.e.m.)**	**Active zone count/ Terminal length. Mean (±s.e.m.)**
***wt***	45.7 (±2.0)	20.3 (±1.0)	109.5 (±4.7)	25.6 (±2.3)	13.5(±1.)	3.3 (±0.3)	1.4 (±0.1)
***spz^2^***	43.9 (±2.2)	30.3 (±4.1)	135.2 (±9.8)	9.4 (±0.9)	5.5 (±0.6)	2.4 (±0.2)	1.1 (±0.1)
***DNT1^41^, DNT2^e03444^***	49.7 (±1.3)	22.2 (±1.4)	131.4 (±5.0)	22.7 (±2.9)	9.4 (±0.7)	2.8 (±0.1)	1.2 (±0.1)
***elavGal4>spzCK***	56.2 (±0.8)	24.1 (±1.9)	133.2 (±6.6)	-	-	-	-
***elavGal4>DNT1CK3'+***	52.6 (±1.2)	25.2 (±1.3)	132.8 (±7.1)	-	-	-	-
***elavGal4>DNT2CK***	47. (±1.2)	22.0 (±2.0)	118.6 (±9.3)	-	-	-	-
***24BGal4>spzCK***	50.4 (±0.8)	25.7 (±1.3)	131.4 (±6.7)	-	-	-	-
***24BGal4>DNT1CK3'+***	46.6 (±1.4)	20.4 (±1.5)	122.9 (±6.8)	-	-	-	-
***24BGal4>DNT2CK***	48.1 (±1.8)	23.8 (±1.3)	128.3 (±4.0)	-	-	-	-
	**Muscle 6,7 NMJ**						
**Genotype**	**MSA µm^2^10^-3^Mean (±s.e.m.)**	**No. of Boutons Mean (±s.e.m.)**	**Terminal length µm Mean (±s.e.m.)**	**DeadEasy Synapse Voxel/ Bouton. mean (±s.e.m.)**	**Active zone count/ Bouton. Mean (±s.e.m.)**	**DeadEasy Synapse Voxel/ Terminal length. Mean (±s.e.m.)**	**Active zone count/ Terminal length. Mean (±s.e.m.)**
***wt***	80.9 (±2.5)	43.9 (±2.0)	290.7 (±20)	37.4 (±5.5)	12.4 (±0.8)	3.3 (±0.2)	1.2 (±0.1)
***spz^2^***	60.7 (±2.3)	37.7 (±6.1)	270.7 (±24)	26.6 (±1.5)	10.9 (±0.6)	3.0 (±0.3)	1.0 (±0.1)
***DNT1^41^, DNT2^e03444^***	69.9 (±2.1)	59.2 (±3.1)	433.7 (±19)	22.4 (±1.3)	8.9 (±0.3)	2.0 (±0.1)	0.8 (±0.03)
***elavGal4>spzCK***	80.9 (±1.6)	51.2 (±3.2)	426.1 (±17)	-	-	-	-
***elavGal4>DNT1CK3'+***	81.7 (±1.8)	57.4 (±5.3)	455.6 (±32)	-	-	-	-
***elavGal4>DNT2CK***	75.3 (±1.7)	50.1 (±3.9)	396.0 (±19)	-	-	-	-
***24BGal4>spzCK***	69.7 (±1.1)	45.5 (±2.6)	301.1 (±12)	-	-	-	-
***24BGal4>DNT1CK3'+***	69.6 (±1.5)	42.2 (±2.2)	308.9 (±12)	-	-	-	-
***24BGal4>DNT2CK***	67.2 (±2.1)	40.7 (±4.0)	321.5 (±22)	-	-	-	-
**Rescue data** Figure **4G,H**							
***wt***	-	-	-	13.2 (±2.6)	16.5 (±1.6)	1.1 (±0.1)	1.2 (±0.1)
***DNT1^41^, Df(3L)6092/DNT1^55^, DNT2^e03444^***	-	-	-	5.2 (±1.1)	11.9 (±0.7)	0.6 (±0.1)	1.0 (±0.03)
***elavGal4>DNT1CK3'+, in DNT1^41^, DNT2^e03444^***	-	-	-	12.3 (±1.6)	19.8 (±1.0)	0.9 (±0.1)	1.2 (±0.04)
***elavGal4>DNT2CK, in DNT1^41^, DNT2^e03444^***	-	-	-	17.6 (±2.1)	22.6 (±1.0)	1.0 (±0.1)	1.3 (±0.1)

**Figure 3:**
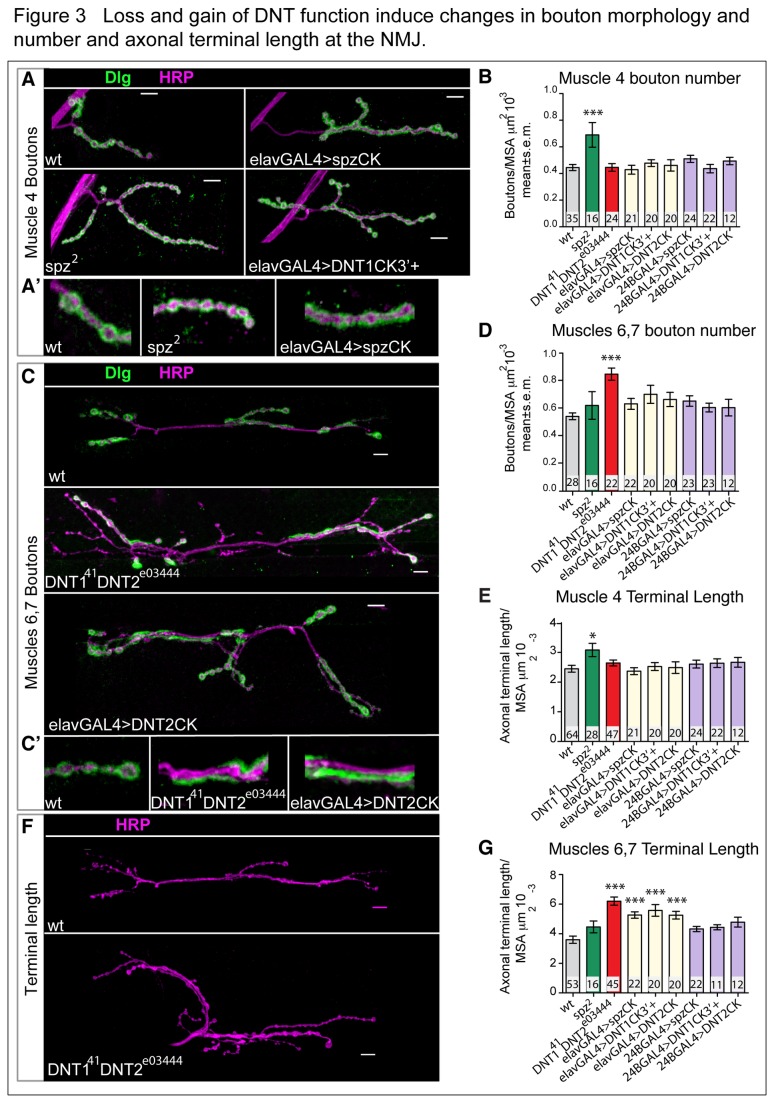
Loss and gain of DNT function induce changes in bouton morphology and number and axonal terminal length at the NMJ. (A,C, F) Third instar larval NMJ preparations, showing synaptic boutons by the colocalisation of pre-synaptic HRP (magenta, also labelling the axons) and post-synaptic Dlg (green). (A’, C’) High magnification details of (A,C) showing an increase in bouton number (A’, *spz* ^2^), and stretches of Dlg signal along the axons with no boutons (A’ *spzCK*, C’) (B,D) Quantification of bouton number normalized to muscle surface area (MSA). (E-G) Axonal terminal length increases upon alterations in DNT function, (F) shows the muscle 6,7 NMJ; quantification in (E,G) shows axonal terminal length normalized over MSA. Numbers within bars indicate n=number of NMJs analysed. Significance: (B,D) One Way ANOVA: p=0.000 and p=0.001, and post-hoc Dunnett corrections. (E) One Way ANOVA: p=0.029. (G) One Way ANOVA: p<0.000. Asterisks indicate comparisons to control yw, Dunnett post-hoc tests, * p<0.05, **p<0.01, ***p<0.001. GAL4 drivers: *elavGAL4* (neurons) or *24BGAL4* (muscle). Scale bars: 20 µm. For raw data see Table 1, for detailed genotypes see Table S1 and for statistical details see Table S2.

Bouton morphology, assessed by Dlg staining, was affected in *spz* ^2^ mutants and in *DNT1* ^*41*^ *, DNT2* ^*e03444*^ double mutant larvae, indicating deficits in post-synaptic structure (Figure 3A’,C’, Table 1). The altered bouton morphology obscured differences between bouton types, thus we quantified all boutons together. In *spz* ^2^ mutant larvae there was a significant increase in bouton number on the muscle 4 NMJ, whereas there was no effect on the muscle 6,7 NMJ (Figure 3A,B,D). Axonal terminal length increased in *spz* ^2^ mutant larvae on muscle 4 but not muscles 6,7 (Figure 3A,E,G). These data show that Spz is normally required for the normal development of the muscle 4 NMJ, but not the muscle 6,7 NMJ. Bouton numbers in *DNT1* ^*41*^ *, DNT2* ^*e03444*^ double mutant larvae were indistinguishable from wild-type at muscle 4, but significantly increased at muscles 6,7 (Figure 3B,C,D). Axon terminal length was normal at the muscle 4 NMJ in *DNT1* ^*41*^ *, DNT2* ^*e03444*^ double mutants, but increased dramatically at the muscle 6,7 NMJ (Figure 3E,F,G). These data show that DNT1 and DNT2 function together preferentially at the muscle 6,7 NMJ.

To ask whether gain of function for *spz, DNT1* and *DNT2* can influence the NMJ, we over-expressed activated forms in either neurons or muscle. Over-expression of *spzCK* in neurons increased axon terminal length at muscle 6,7 NMJ, but did not affect the muscle 4 NMJ (Figure 3A,D, Table 1). This shows that Spz can influence the muscle 6,7 NMJ, although it does not normally do so in wild-type. Over-expression of *DNT1CK3*’*+* or *DNT2CK* in all neurons increased terminal length only at muscles 6,7 (Figure 3B,C,D,E,G), showing that DNT1 and DNT2 can promote muscle 6,7 NMJ growth. Over-expression of *spzCK, DNT1CK3*’*+* or *DNT2CK* in muscles had no effect (Figure 3B,D,E,G). In *DNT1* ^*41*^ *, DNT2* ^*e03444*^ double mutants and upon expression of activated forms of the DNTs, we observed aberrant post-synaptic Dlg distribution alongside the axonal terminals, rather than forming discrete boutons (Figure 3C,C’). This phenotype reflects a breakdown of the post-synaptic structure.

Altogether, these data show that Spz function is required for the normal development of the muscle 4 NMJ, and DNT1 and DNT2 for that of muscle 6,7 NMJ. The fact that Spz can also affect the muscle 6,7 NMJ suggests that the DNTs may possibly be promiscuous ligands binding similar receptor types. The DNTs are secreted proteins, but we have no evidence that their over-expression results in secretion. Thus, the stronger effects obtained expressing them in neurons might reflect functional activity in the expressing cells only. Alternatively, it could also suggest that retrograde protein take up or transport from muscle is sub-threshold, or that neuronal production of the ligands improves their accessibility to the receptors, or that the DNTs may have dendritic, anterograde or autocrine functions.

### Altered DNT function affects active zones and results in synaptic debris and ghost boutons

We next tested whether active zones, the pre-synaptic sites of synaptic vesicle docking for neurotransmitter release, were affected in the mutants. We visualised active zones with anti-nc82/Bruchpilot (Brp) and both measured the total volume of nc82/Brp signal in confocal stacks of images using purposely-developed automatic DeadEasy Synapse software and also counted nc82/Brp spots manually (Table 1). DeadEasy Synapse counts the total voxel occupancy by the nc82/Brp signal, which is very discrete, strong and devoid of non-specific background.

**Figure 4:**
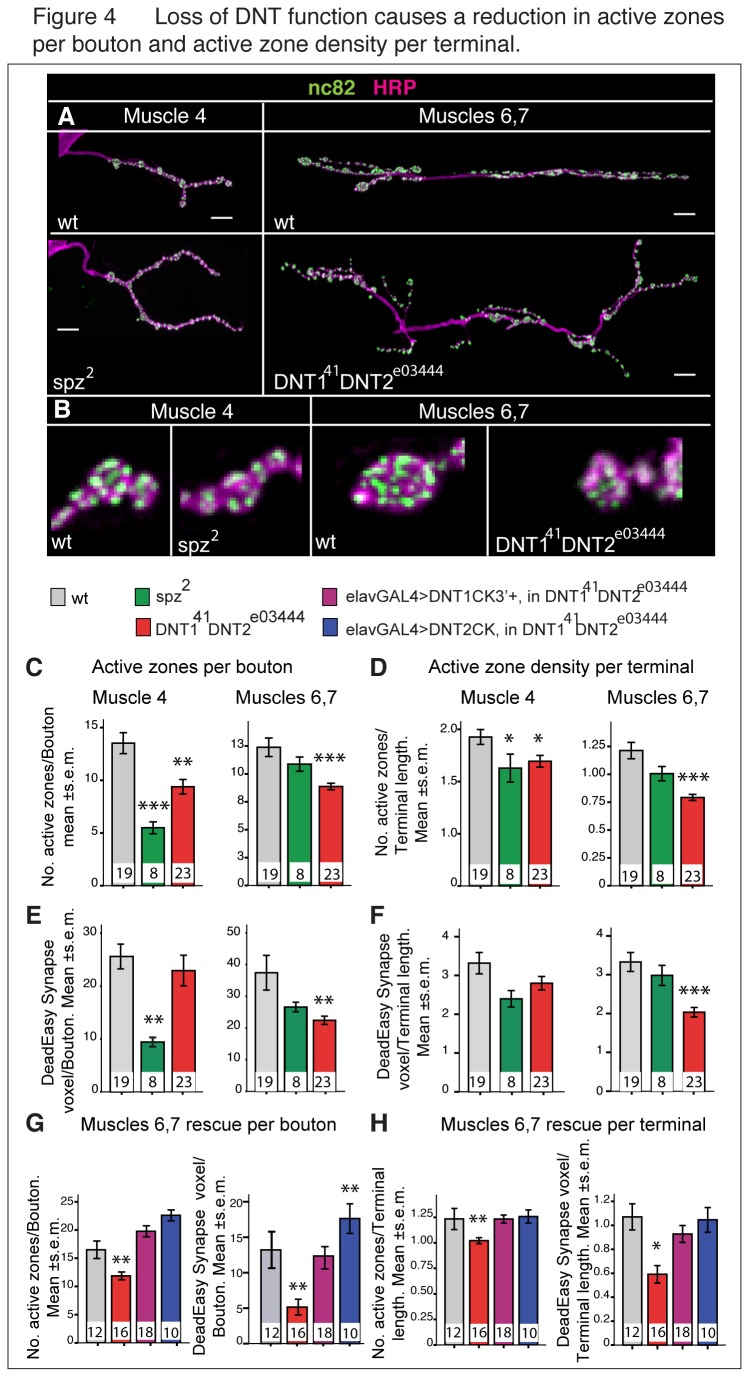
Loss of DNT function causes a reduction in active zones. (A,B) Third instar larval NMJ preparations showing active zones labelled with nc82/Brp (green) over HRP+ axons (magenta). (B) There is a reduction in active zones per bouton in the mutants (higher magnification details from A). (C, E) Number of active zones per bouton: (C) counted manually and (E) using DeadEsy Synapse (as total nc82/Brp voxel occupancy per bouton), one bouton was chosen randomly from each NMJ. (D, F) Number of active zones per NMJ terminal: (D) counted manually and (F) active zone density per NMJ terminal, measured using DeadEasy Synapse (as total nc82/Brp voxel occupancy per terminal), decreases in the mutants. (G) Rescue: the reduction in active zone number per bouton and DeadEasy voxel volume per bouton at the muscle 6,7 NMJ in *DNT1* ^*41*^ *DNT2* ^*e03444*^ double mutants is rescued with the over-expression of the *DNTs* in neurons. (H) Rescue: the number of active zones per NMJ terminal and of total active zone occupancy in the 6,7 NMJ in *DNT1* ^*41*^ *DNT2* ^*e03444*^ double mutants is rescued with the over-expression of the *DNTs* in neurons. Numbers within bars indicate n=number of NMJs analysed. Asterisks indicate comparisons to *yw* control, all samples were analysed using One Way ANOVA and post-hoc Dunnett tests: *p<0.05, **p<0.01, ***p<0.001. Scale bars: 20 µm. For raw data see Table 1, for detailed genotypes see Table S1 and for statistical details see Table S2.

Compared to wild-type, *spz* ^2^ mutants showed a significant reduction in the number of active zones per bouton at the muscle 4 NMJ but not at the muscle 6,7 NMJ (Figure 4A,B,C,E). In *DNT1* ^*41*^ *, DNT2* ^*e03444*^ double mutants there was a significant reduction in the number of active zones per bouton at the muscle 6,7 NMJ but not at the muscle 4 NMJ (Figure 4A,B,C,E). These data show that there is a reduction in the number of active zones in the mutants at specific NMJs.

To test whether this reduction was reflected in the density of active zones per NMJ terminal, we counted the total number of active zones in each NMJ with the same methods as above, and normalised the counts relative to NMJ terminal length. Active zone density throughout the terminal was lower at the muscle 4 NMJ, but not muscle 6,7 NMJ, in *spz* ^2^ mutants (Figure 4A,D,F), and was lower at the muscle 6,7 NMJ, but not muscle 4 NMJ, in *DNT1* ^*41*^ *, DNT2* ^*e03444*^ mutants (Figure 4A,D,F).

The reduction in the number of active zones per bouton at the muscle 6,7 NMJ was reproduced in *DNT1* ^*55*^ *, DNT2 e03444*/*DNT1* ^*41*^ *, Df*(*3L*)*6092* trans-heterozygous double mutants (Figure 4G) and it was rescued with the over-expression of either *DNT1CK3*’*+* or *DNT2CK* (Figure 4G). Total active zone volume in the muscle 6,7 NMJ terminal decreased in *DNT1* ^*55*^ *, DNT2 e03444*/*DNT1* ^*41*^ *, Df*(*3L*)*6092* double mutants (Figure 4H) and this was rescued with the over-expression of either *DNT1CK3*’*+* or *DNT2CK* (Figure 4H). These data demonstrate that in these mutants the deficit in active zones is due to the loss of DNT1 and DNT2 function. Altogether, these data show a deficit in active zone generation or stability in the mutants. The data also confirm that Spz function is required for the muscle 4 NMJ, and that the combined function of DNT1 and DNT2 is required for the muscle 6,7 NMJ.

**Figure 5:**
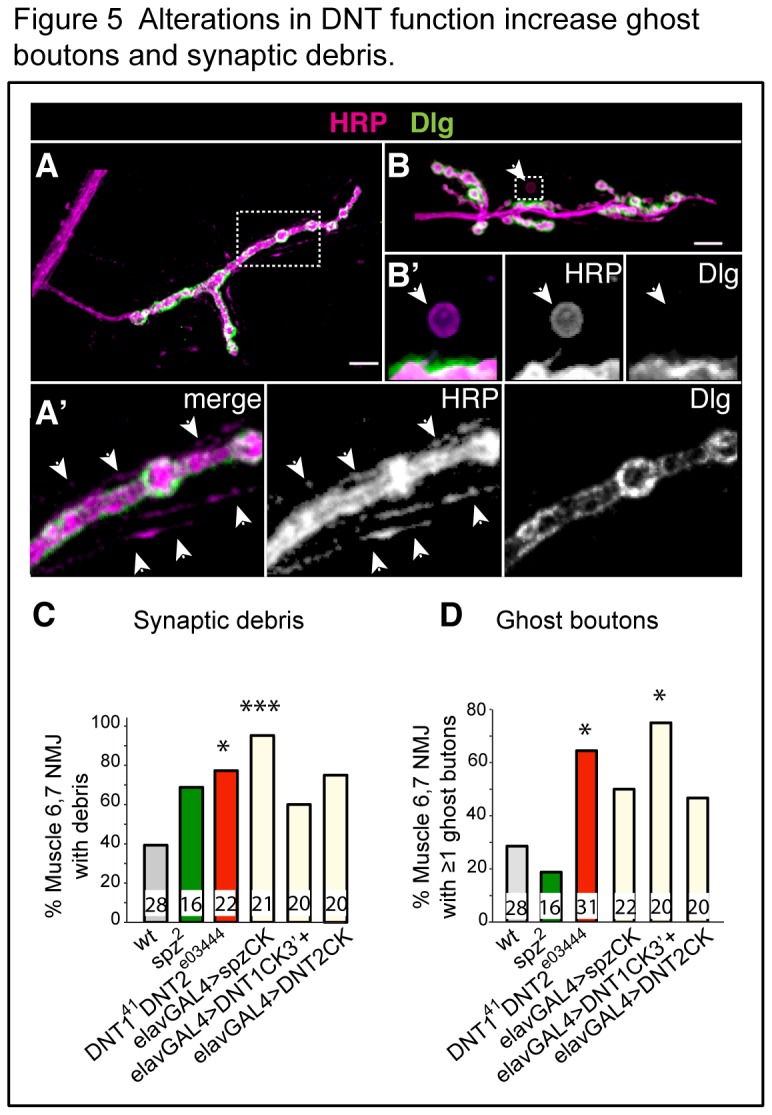
Alterations in DNT function increase ghost boutons and synaptic debris. (A,A’) Synaptic debris is pre-synaptic HRP+ but post-synaptic Dlg- cellular material along the terminals. (B,B’) Ghost boutons (arrowheads) are pre-synaptic HRP+ and post-synaptic Dlg -. (A’,B’) are higher magnification details. (C, D) Manual quantification. Numbers within bars indicate n=number of NMJs analysed. Chi-square tests, *p<0.05, **p<0.01, ***p<0.001. GAL4 drivers: *elavGAL4* (neurons) and *24BGAL4* (muscle). Scale bars: 20 µm. For detailed genotypes see Table S1 and for statistical details see Table S2.

The incidence of ghost boutons and synaptic debris increases with neuronal activity [34-36]. Synaptic debris is shed pre-synaptic membrane (stains HRP positive) during NMJ growth, and it is normally cleared by glial cells [36] and muscle [32]. Ghost boutons are nascent boutons that lack both post-synaptic differentiation and presynaptic active zones, and are positive for HRP (pre-synaptic) but lack Dlg (post-synaptic) signal, and are cleared by muscle [34-36]. In *DNT1* ^*41*^ *, DNT2* ^*e03444*^ double mutant larvae there was an increase in the incidence of synaptic debris at both the muscle 4 (data not shown) and muscle 6,7 NMJs (Figure 5A,C). Over-expression of *spzCK* in all neurons increased synaptic debris at the muscle 6,7 NMJ (Figure 5C). In *DNT1* ^*41*^ *, DNT2* ^*e03444*^ double mutant larvae there was an increase in the incidence of ghost boutons at the muscle 6,7 NMJ (Figure 5B,D) and over-expression of *DNT1CK3*’*+* caused a significant increase in ghost boutons at the muscle 6,7 NMJ (Figure 5B,D). These findings suggest that Spz, DNT1 and DNT2 positively influence synaptic activity, or that interference with their function affects clearance of synaptic material.

### Reduced mEJP frequency and amplitude at the *spz* ^2^ mutant NMJ

**Figure 6:**
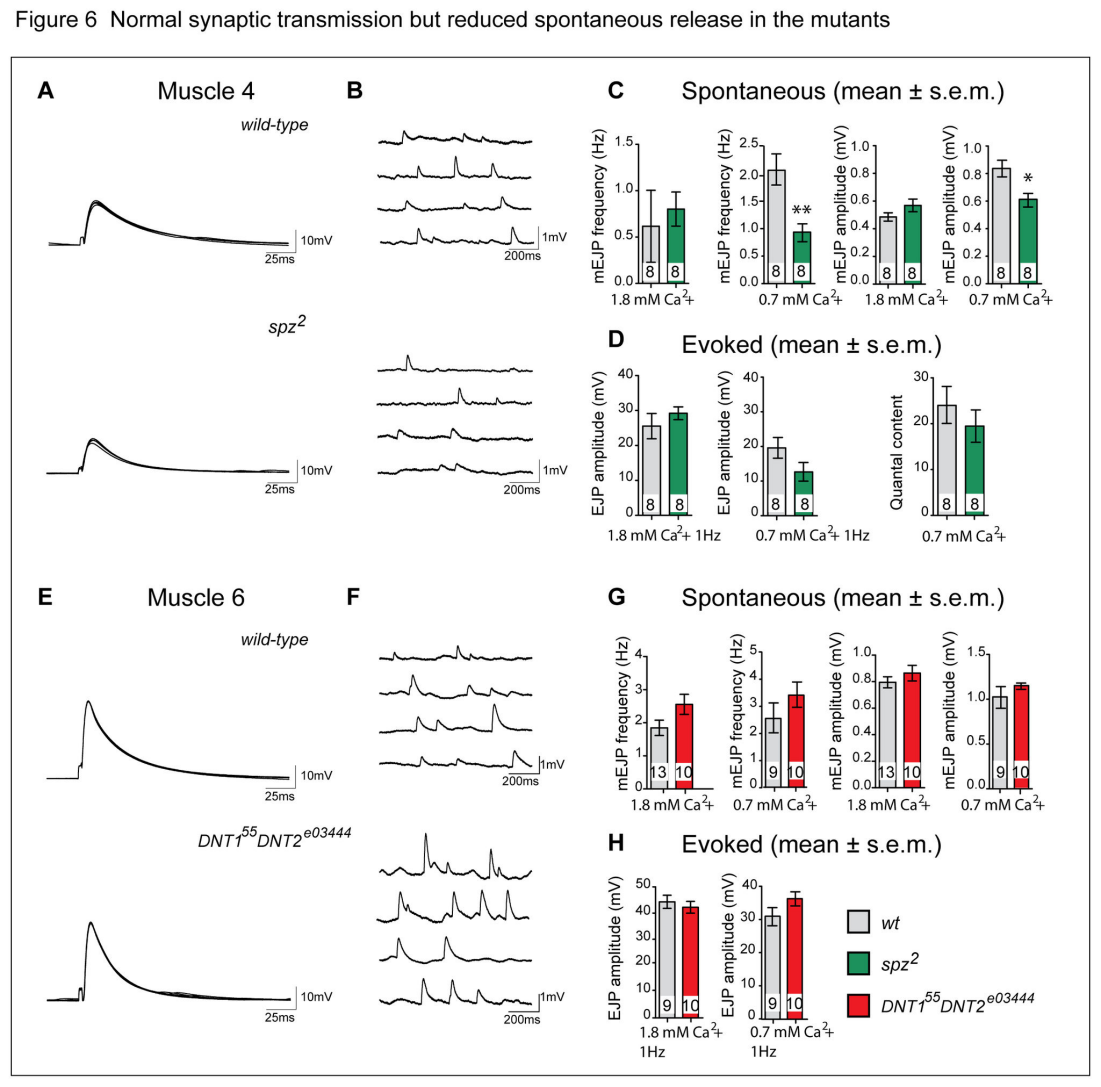
Normal synaptic transmission, but reduced spontaneous release in the mutants. (A,E) Five representative traces showing evoked excitatory junction potentials (EJPs) evoked by 1 Hz stimulation and low extracellular calcium, recorded from muscle 4, 6 or 7 of wild-type (yw), *spz* ^2^ and *DNT1* ^*55*^ *DNT2* ^*e03444*^ mutant third instar larvae. Loss of *spz, DNT1* and *DNT2* has no effect on either the time course (A,E) or the amplitude (D,H) of the response. (B,F) Four representative traces of recordings of spontaneous neurotransmitter release (mEJPs). (C,G) The frequency and amplitude of the spontaneous mEJPs recorded from muscle 4 are both reduced in *spz* ^2^ mutants; quantal content is not affected (D). Spontaneous mEJPs are not affected in muscle 6,7 (G). Student-t tests for pair-wise comparisons, *p<0.05, **p<0.01, ***p<0.001. For detailed genotypes see Table S1 and for statistical details see Table S2.

We next asked whether synaptic transmission was affected in the mutants. Excitatory junction potentials (EJPs) from abdominal muscles of wandering third instar larvae were recorded in normal and low extracellular calcium conditions. We recorded from the second or third abdominal segment (A2 or A3) from muscle 4 in *spz* ^2^ mutants, and from muscle 6 or 7 in *DNT1* ^*55*^ *, DNT2* ^*e03444*^ double mutants. In *spz* ^2^ mutants in low extracellular calcium revealed a significant reduction in mEJP frequency and amplitude (Figure 6B,C). Evoked EJP amplitude was unaffected in *spz* ^2^ mutants, under normal or low calcium levels, and quantal content did not change (Figure 6A,D). Nevertheless, the mild albeit not significant trend to a reduced evoked EJP and quantal content in *spz* ^2^ mutants might suggest a decrease in vesicle content or size, altered synaptic vesicle distribution, or deficient clustering of GluRs. Functional compensation by other DNTs and/or homeostatic compensation may conceal a potentially more severe evoked phenotype.

In *DNT1* ^*55*^ *, DNT2* ^*e03444*^ double mutants there was no difference in mEJP frequency or amplitude at muscles 6,7 compared to wild-type, even in low extracellular calcium (Figure 6F,G). Evoked EJP amplitude was also unaffected (Figure 6E,H). However, we have so far been unsuccessful at generating a *DNT2* null mutant, therefore it is still possible that the complete absence of DNT2 might uncover an electrophysiological phenotype. Alternatively, the normal evoked EJP in *DNT1* ^*55*^ *, DNT2* ^*e03444*^ mutants suggests that homeostatic mechanisms may compensate for synaptic deficits by increasing NMJ size, thus restoring normal synaptic transmission.

## Discussion

In summary, our data show that the Drosophila NTs DNT1, DNT2 and Spz are produced in muscles, required at the larval NMJ synapse with neuron type specificity, that alterations in their function affect synaptic structure and, at least in the case of Spz, also physiology. The *spz* ^2^ allele is a mutation in the pro-domain that interferes with the secretion of Spz in cell culture [13]. Our data show that the semi-lethality of the *spz* ^2^ allele can be rescued with the over-expression of activated *Toll* ^*10b*^ in neurons implying that the *spz* ^2^ mutation causes a reduction in normal *spz* function. We do not currently understand the mechanism by which the *spz* ^2^ allele affects function, and this is a question that we aim to solve in the future. The *DNT1* ^41^ and *DNT1* ^55^ mutant alleles are null and produce no protein. *DNT2* ^*e03444*^ is a Piggy-Bac insertion allele that is hypomorphic. We have been unable to generate a *DNT2* null allele, thus it is conceivable that a null might have revealed more dramatic phenotypes. We showed that *DNT1, DNT2* and *spz* are expressed in the larval body wall muscle, and we used the temperature sensitive semi-lethality of the mutants to address the question of whether the DNTs might be functional in neurons. Our data showed that over-expression of *DNT1CK*’*+, DNT2CK* and *spzCK* restricted to neurons can rescue the semi-lethality of the mutants. We did not hypothesise a link between the larval muscle expression and adult survival, and it does not necessarily exist. Our data show importantly that DNT1, DNT2 and Spz can function in neurons and that these neuronal functions are essential for viability.

Reduced Spz, DNT1 and DNT2 function increased terminal size and bouton number, caused abnormal post-synaptic bouton morphology, reduced number of active zones per bouton, reduced active zone density per NMJ terminal and increased shedding of synaptic material. The deficit in active zones in *DNT1 DNT2* double mutants could be rescued by the over-expression of either *DNT1CK3*’*+* or *DNT2CK* in neurons, demonstrating that this phenotype was the direct result of loss of *DNT1* and *DNT2* function. Our experiments did not reveal rescue of the *spz* ^2^ phenotype, which could be due to technical reasons or to the nature of *spz* ^2^ allele.

To ease the analysis, we also developed an automatic method to quantify anti-Brp (nc82) staining at the NMJ, which we named DeadEasy Synapse. We present data acquired both using conventional manual counting and DeadEasy Synapse. Both methods revealed the same results, thus validating DeadEasy Synapse, which will be of great use to the Drosophila NMJ community. The plug-in works with ImageJ and will be made publicly available through our lab webpage.

Aberrant Spz function in the *spz* ^2^ allele resulted in reduced frequency and amplitude of spontaneous mEJPs. However, evoked EJPs were normal for all mutants examined.

Altogether, our data strongly suggest that DNT1, DNT2 and Spz are required for synaptogenesis. They also suggest that the increase in NMJ terminal size and bouton number in the mutants corresponds to a homeostatic structural adjustment that compensates for the reduced number of active zones, thus restoring the overall number of functional release sites and synaptic transmission. Remarkably, Spz functions at the muscle 4 NMJ, whereas the combined functions of DNT1 and DNT2 are required for the muscle 6,7 NMJ.

### DNTs are retrograde factors that influence pre-synaptic function

It has long been known that homeostatic compensatory mechanisms adjust the NMJ terminal to muscle size as the larva grows, to maintain synaptic efficacy within an appropriate physiological range [33]. Increased synaptic growth is accompanied by a decrease in transmitter release per bouton resulting in normal muscle excitation [34,35,36]. Conversely, mutants with fewer boutons have normal physiology, as each bouton has more active zones maintaining a constant overall number per NMJ [37,38]. An analogous scenario is seen in mammals: in NT4 knockout mice, a reduction in post-synaptic AChR density induces a compensatory increase in NMJ terminal area [39]. In Drosophila, it has long been anticipated that retrograde factors produced in the muscle regulate pre-synaptic neurotransmitter release, perhaps by regulating the number of presynaptic active zones in each bouton or some aspect of the presynaptic release mechanism, but their discovery has been scarce [2]. One retrograde growth factor at the Drosophila NMJ is Gbb [3].

Our data are consistent with the DNTs functioning as retrograde growth factors. Firstly, they are expressed at the muscle, and muscle over-expression of *spz* and *DNT2* can rescue the semi-lethality of *spz* ^2^ mutants. Secondly, activated Toll rescues the semi-lethality of *spz* ^2^ mutants when expressed in neurons but not in muscle. Thirdly, *DNT2* transcripts are localised at the boutons and over-expression of *DNT2CK* in neurons rescues the semi-lethality of *DNT1* ^*41*^ *, DNT2* ^*e0344*^ double mutants. However, further evidence that over-expressed cleaved *spzCK, DNT1CK3*’*+* and *DNT2CK* using the GAL4 system can be secreted and taken up normally by receiving cells would be desirable. There is robust evidence that *spzCK, DNT1CK3*’*+* and *DNT2CK* are functional cell-autonomously in the cells in which they are over-expressed using the GAL4/UAS system [11,19,20]. However, inappropriate secretion upon over-expression of the cleaved forms could explain why over-expression from muscle is not as effective as over-expression from neurons at rescuing the semi-lethality of the mutants.

Autocrine and anterograde functions of the DNTs in neurons and/or muscle are also likely. Toll is expressed in muscle where it functions as an inhibitor of synaptogenesis [24,25,26,27]. Targeting by motoraxons coincides with a downregulation of Toll at the target muscle, and over-expression of Toll in muscle reduces bouton number at the muscle 6,7 NMJ [27]. The DNTs may also have bidirectional functions. *DNT2* transcripts are localised post-synaptically, reminiscent of the post-synaptic expression of *BDNF* and *NT4*. Their transcripts are translated in response to neuronal activity and the combination of anterograde and retrograde functions results in synaptic potentiation [44]. DNTs, perhaps particularly DNT2, may also have bidirectional functions at the synapse. The full characterisation of retrograde and/or anterograde functions of the DNTs must await the discovery of the receptors functioning at the NMJ, and the production of good antibodies that enable visualisation of the endogenous ligands.

Our data indicate that the DNTs are required for synaptogenesis. DNT loss of function mutants display increased bouton number and increased terminal size but reduced number of active zones per bouton and normal EJPs. Most likely, the increase in terminal size and bouton number is a homeostatic compensation for the deficient formation and function of active zones. In *DNT1* ^*41*^ *, DNT2* ^*e0344*^ mutants, muscles are smaller compared to wild-type and active zone density is reduced, but the NMJ is larger. This suggests that since in *DNT* mutants boutons have fewer synapses, the NMJ expands making more boutons, thus compensating for the synaptic deficits and maintaining overall normal function. Over-expression of DNTs in neurons increases muscle size, ghost boutons and shed synaptic debris, phenotypes that are consistent with a function of the DNTs in promoting axonal and muscle growth and/or in synaptic transmission. On the other hand, the observation that loss and gain of *DNT* function results in increased production of synaptic debris and ghost boutons, could also reflect defective clearance of synaptic material (e.g. by muscle or glia) upon interference with DNT function.

In *spz* ^2^ mutants in physiological calcium levels no changes in synaptic transmission were observed; lowering the extracellular calcium concentration reveals reduced frequency and amplitude of mEJPs, correlating with a reduction in the number of active zones per bouton. Despite the aberrant frequency and amplitude of spontaneous mEJPs in *spz* ^2^ mutants in low calcium, evoked EJPs were normal under all conditions for all mutants examined and loss of DNTs did not affect synaptic transmission.

These phenotypes are reminiscent of those found upon manipulation of another invertebrate neurotrophin superfamily member in mollusks, *Aplysia* neurotrophin (ApNT) [40]. Alterations in ApNT levels influence synaptic structure and the formation of synaptic varicosities. Expression of a dominant negative form of the receptor Ap-Trk-DN in cultured neurons had no effect in synaptic transmission following a single pulse of 5-HT, and effects were only seen in Long Term Facilitation after 5 consecutive pulses [40]. Similarly, expression of ApNT or bathing cells in ApNT also led to more pronounced increases in evoked potential in Long Term Facilitation [40]. Conceivably, high frequency stimulation might reveal enhanced effects in synaptic transmission upon manipulation of DNT function, and this is something worth testing in the future.

To conclude, in the case of Drosophila, it is most likely that the increase in NMJ terminal size and bouton number in the DNT mutants corresponds to a homeostatic structural adjustment that compensates for the reduced number of active zones, restoring the overall number of functional release sites and subsequent synaptic transmission.

### DNTs function with neuron-type specificity at the NMJ

The DNTs are the first growth factors to be identified to have neuron-type specificity at the Drosophila NMJ: the *spz* ^2^ mutation affected the muscle 4 NMJ, and *DNT1* ^*41*^ *, DNT2* ^*e0344*^ double mutants affected the muscle 6,7 NMJ. This observed neuron-type specificity is reminiscent of the neuronal modality of mammalian NT and Trk receptor function in the central and peripheral nervous systems [41,42,43,44]. However, we only analysed the muscles 4 and 6,7 NMJs, not all muscles in the larva, thus we cannot rule out the possibility that the DNTs may have more redundant and less specific functions in other muscles. How the observed specificity comes about is not yet understood, since our current evidence indicates that the three ligands are expressed throughout the muscles. In future work, antibodies to the DNTs may provide higher resolution and reveal distinct distribution patterns. For now, the distribution of DNT2 transcripts in synaptic boutons is a significant difference. In any case, our data also show that there is some functional redundancy between Spz, DNT1 and DNT2. DNTs may be promiscuous ligands that in some circumstances (e.g. upon over-expression) can bind multiple receptors, and thus neurons may be able to respond to the excess of any of the DNTs. This is reminiscent of the redundancy between vertebrate NT ligands, and the fact that different NTs can bind the same Trk receptor [41,42,43,44]. In the normal larva, the distinct NMJ-specific effects may reflect the distribution of the DNT receptors in distinct neuronal types, and testing this hypothesis will await the identification of the receptors for DNT1 and DNT2. In any case, the NMJ specificity precisely reflects the neuron type specificity for the DNTs that also takes place during motoraxon targeting in the embryo: DNT1 is required for targeting of ISNb/d and Spz for targeting of SNa motoraxons [11]. Intriguingly, since specific DNTs appear to be required for both motor-axon targeting and synapse formation at the NMJ, this would suggest that such neuron-type specific functions serve to shape motor-neuron-muscle connectivity required for the organisation of locomotor behaviour. Future progress will tackle the identification of the DNT receptors, the basis of this neuron-type specificity and the relevance for behaviour.

## Supporting Information

Table S1 **Genotypes.**
(DOCX)

Table S2 **Statistical analysis.**
(DOCX)
