# Supplementary material for: Neuron-Type Specific Functions of DNT1, DNT2 and Spz at the Drosophila Neuromuscular Junction
Source: PLoS One. 2013 Oct 4;8(10):e75902. doi: 10.1371/journal.pone.0075902 (PMC3790821; doi:10.1371/journal.pone.0075902)
Supplement: Table S1 — Genotypes. (DOCX) [file pone.0075902.s001.docx]

Supplementary Table S1 Genotypes

| **Figure** | **Genotype** | **Referred to as** |
| --- | --- | --- |
| 2A | spz^2^, ca^1^/TM6BlacZ | spz^2^ |
| 2A | elavGAL4, spz^2^/TM6BlacZ | elavGAL4, in spz^2^ |
| 2A | 24BGAL4, spz^2^/TM6BlacZ | 24BGAL4, in spz^2^ |
| 2A | UAS-p35; spz^2^/TM6BlacZ | UASp35, in spz^2^ |
| 2A | UAS-spzCysknot/ UAS-spzCysknot; spz^2^/TM6BlacZ | UASspzCK, in spz^2^ |
| 2A | UAS-Toll^10b^/ UAS-Toll^10b^; spz^2^/TM6BlacZ | UASToll^10b^, in spz^2^ |
| 2A | UAS-DNT1Cysknot3’+3E, spz^2^/TM6BlacZ | UASDNT1CK3'+, in spz^2^ |
| 2A | UAS-DNT2Cysknot 6B, spz^2^/TM6BlacZ | UASDNT2CK, in spz^2^ |
| 2A | 24BGAL4, spz^2^/TM6BlacZ | 24BGAL4, in spz^2^ |
| 2A | UAS-p35/+; elavGAL4, spz^2^/spz^2^ | elavGAL4>p35, in spz^2^ |
| 2A | UAS-spz^14^/+; elavGAL4, spz^2^/spz^2^ | elavGAL4>spzCK, in spz^2^ |
| 2A | UAS-Toll^10b^/+; elavGAL4, spz^2^/spz^2^ | elavGAL4>Toll^10b^, in spz^2^ |
| 2A | UAS-DNT1Cysknot3’+3E, spz^2^/elavGAL4, spz^2^ | elavGAL4>DNT1CK3'+, in spz^2^ |
| 2A | UAS-DNT2Cysknot 6B, spz^2^/elavGAL4, spz^2^ | elavGAL4>DNT2CK, in spz^2^ |
| 2A | UAS-spz/+; 24BGAL4, spz^2^/spz^2^ | 24BGAL4>spzCK, in spz^2^ |
| 2A | UAS-Toll^10b^/+; 24BGAL4, spz^2^/spz^2^ | 24BGAL4>Toll10b, in spz^2^ |
| 2A | UAS-DNT1Cysknot3’+3E, spz^2^/24BGAL4, spz^2^ | 24BGAL4>DNT1CK3'+, in spz^2^ |
| 2A | UAS-DNT2Cysknot 6B, spz^2^/24BGAL4, spz^2^ | 24BGAL4>DNT2CK, in spz^2^ |
| 2B | DNT1^41^, DNT2^e03444^/TM6BlacZ | DNT1^41^DNT2^e03444^ |
| 2B | DNT1^41^, Df(3L)Exel6092/DNT1^41^, DNT2^e03444^ | DNT1^41^Df(3L)6092/DNT1^41^DNT2^e03444^ |
| 2B | elavGAL4, DNT1^41,^ DNT2^e03444^/TM6BlacZ | elavGAL4, in DNT1^41^DNT2^e03444^ |
| 2B | chaGAL4/ chaGAL4; DNT1^41^, DNT2^e03444^/TM6BlacZ | chaGAL4, in DNT1^41^DNT2^e03444^ |
| 2B | 24BGAL4, DNT1^41^, DNT2^e03444^/TM6BlacZ | 24BGAL4, in DNT1^41^DNT2^e03444^ |
| 2B | UAS-p35/ UAS-p35; DNT1^41^, DNT2^e03444^/TM6BlacZ | UASp35, in DNT1^41^DNT2^e03444^ |
| 2B | UAS-DNT1Cysknot3'+3E, DNT1^41^, DNT2^e03444^/TM6BlacZ | UASDNT1CK3'+, in DNT1^41^DNT2^e03444^ |
| 2B | UAS-DNT2cysknot6B, DNT1^41^, DNT2^e03444^/TM6BlacZ | UASDNT2CK, in DNT1^41^DNT2^e03444^ |
| 2B | UAS-EP(X)1516;; DNT1^41^, DNT2^e03444^/TM6BlacZ | UASdTRAF2, in DNT1^41^DNT2^e03444^ |
| 2B | UAS-spz^Cysknot^; DNT1^41^, DNT2^e03444^/TM6BlacZ | UASspzCK, in DNT1^41^DNT2^e03444^ |
| 2B | DNT1^41^DNT2^e03444^elavp35 9.1/TM6BlacZ | DNT1^41^,DNT2^e03444^, elavp35 9.1, in DNT1^41^DNT2^e03444^ |
| 2B | chaGAL4/UAS-p35; DNT1^41^, DNT2^e03444^/DNT1^41^, DNT2^e03444^ | chaGAL4>p35, in DNT1^41^DNT2^e03444^ |
| 2B | UAS-p35/+; elavGAL4, DNT1^41^, DNT2^e03444^/DNT1^41^, DNT2^e03444^ | elavGAL4>p35, in DNT1^41^DNT2^e03444^ |
| 2B | elavGAL4, DNT1^41^, DNT2^e03444^/UAS-DNT1Cysknot3'+3E, DNT1^41^, DNT2^e03444^ | elavGAL4>DNT1CK3'+, in DNT1^41^DNT2^e03444^ |
| 2B | elavGAL4, DNT1^41^, DNT2^e03444^/UAS-DNT2Cysknot 6B, DNT1^41^, DNT2^e03444^ | elavGAL4>DNT2CK, in DNT1^41^DNT2^e03444^ |
| 2B | elavGAL4, DNT1^41^, DNT2^e03444^/UAS-spz, DNT1^41^, DNT2^e03444^ | elavGAL4>spzCK, in DNT1^41^DNT2^e03444^ |
| 2B | UAS-EP(X)1516/+ or Y;; elavGAL4, DNT1^41^, DNT2^e03444^/DNT1^41^, DNT2^e03444^ | elavGAL4>dTRAF2, in DNT1^41^DNT2^e03444^ |
| 2B | UAS-EP(X)1516/+ or Y; chaGAL4/+; DNT1^41^, DNT2^e03444^/DNT1^41^, DNT2^e03444^ | chaGAL4>dTRAF2, in DNT1^41^DNT2^e03444^ |
| 2B | 24BGAL4, DNT1^41^, DNT2^e03444^/UAS-DNT2Cysknot 6B, DNT1^41^, DNT2^e03444^ | 24BGAL4>DNT2CK, in DNT1^41^DNT2^e03444^ |
| 3B, C, D, E | yw | wt |
| 3B, C, D, E | spz^2^, ca^1^ | spz^2^ |
| 3B, C, D, E | DNT1^41^, DNT2^e03444^ | DNT1^41^DNT2^e03444^ |
| 3B, C, D, E | UAS-spz/+; elavGAL4, spz^2^/spz^2^ | elavGAL4>spzCK |
| 3B, C, D, E | UAS-DNT1Cysknot3’+3E, spz^2^/elavGAL4, spz^2^ | elavGAL4>DNT1CK3'+ |
| 3B, C, D, E | UAS-DNT2Cysknot 6B, spz^2^/elavGAL4, spz^2^ | elavGAL4>DNT2CK |
| 3B, C, D, E | UAS-spzCysknot/+; 24BGAL4, spz^2^/spz^2^ | 24BGAL4>spzCK |
| 3B, C, D, E | UAS-DNT1Cysknot3’+3E, spz^2^/24BGAL4, spz^2^ | 24BGAL4>DNT1CK3'+ |
| 3B, C, D, E | UAS-DNT2Cysknot 6B, spz^2^/24BGAL4, spz^2^ | 24BGAL4>DNT2CK |
| 4C, D, E, F | yw | wt |
| 4C, D, E, F | spz^2^, ca^1^ | spz^2^ |
| 4C, D, E, F | DNT1^41^, DNT2^e03444^ | DNT1^41^DNT2^e03444^ |
| 4G, H | yw | wt |
| 4G, H | DNT1^55^, DNT2^e03444^/DNT1^41^,Df(3L)Exel6092 | DNT1^55^DNT2^e03444^ |
| 4G, H | elavGAL4, DNT1^41^, DNT2^e03444^/UAS-DNT1Cysknot3'+3E, DNT1^41^, DNT2^e03444^ | elavGAL4>DNT1CK3'+, in DNT1^41^,DNT2^e03444^ |
| 4G, H | elavGAL4, DNT1^41^, DNT2^e03444^/UAS-DNT2Cysknot 6B, DNT1^41^, DNT2^e03444^ | elavGAL4>DNT2CK, in DNT1^41^,DNT2^e03444^ |
| 5C, D | yw | wt |
| 5C, D | spz^2^, ca^1^ | spz^2^ |
| 5C, D | DNT1^41^, DNT2^e03444^ | DNT1^41^DNT2^e03444^ |
| 5C, D | UAS-spzCysknot/+; elavGAL4, spz^2^/spz^2^ | elavGAL4>spzCK |
| 5C, D | UAS-DNT1Cysknot3’+3E, spz^2^/elavGAL4, spz^2^ | elavGAL4>DNT1CK3'+ |
| 5C, D | UAS-DNT2Cysknot 6B, spz^2^/elavGAL4, spz^2^ | elavGAL4>DNT2CK |
| 6C, D, G, H | yw | wt |
| 6C, D, G, H | spz^2^, ca^1^ | spz^2^ |
| 6C, D, G, H | DNT1^55^, DNT2^e03444^ | DNT1^55^DNT2^e03444^ |
