# Supplementary material for: Neuron-Type Specific Functions of DNT1, DNT2 and Spz at the Drosophila Neuromuscular Junction
Source: PLoS One. 2013 Oct 4;8(10):e75902. doi: 10.1371/journal.pone.0075902 (PMC3790821; doi:10.1371/journal.pone.0075902)
Supplement: Table S2 — Statistical analysis. (DOCX) [file pone.0075902.s002.docx]

Supplementary Table S2 Statistical analysis

| **Figure** | **Test used** | **Software** | **Comparison** | **n** | **P value** | **Significance** |
| --- | --- | --- | --- | --- | --- | --- |
|  | Chi-Square | SPSS 21 | Survival index: all genotypes |  | 0.000 | *** |
| 2A | Chi-Square, Bonferroni | SPSS 21 | Survival index: elavGal4, in spz^2^ vs. elavGal4>p35, in spz^2^ | 236, 94 | 0.666 | ns |
| 2A | Chi-Square, Bonferroni | SPSS 21 | Survival index: elavGal4, in spz^2^ vs. elavGal4>spzCK, in spz^2^ | 236, 124 | 0.000 | *** |
| 2A | Chi-Square, Bonferroni | SPSS 21 | Survival index: elavGal4, in spz^2^ vs. elavGal4>Toll^10b^, in spz^2^ | 236, 117 | 0.000 | *** |
| 2A | Chi-Square, Bonferroni | SPSS 21 | Survival index: elavGal4, in spz^2^ vs. elavGal4>DNT1CK3’+, in spz^2^ | 236, 171 | 0.000 | *** |
| 2A | Chi-Square, Bonferroni | SPSS 21 | Survival index: elavGal4, in spz^2^ vs. elavGal4>DNT2CK, in spz^2^ | 236, 211 | 0.000 | *** |
| 2A | Chi-Square, Bonferroni | SPSS 21 | Survival index: 24bGal4, in spz^2^ vs. 24BGal4>spzCK, in spz^2^ | 1283, 368 | 0.000 | *** |
| 2A | Chi-Square, Bonferroni | SPSS 21 | Survival index: 24BGal4, in spz^2^ vs. 24BGal4>Toll^10b^, in spz^2^ | 1283, 229 | 6.354 | ns |
| 2A | Chi-Square, Bonferroni | SPSS 21 | Survival index: 24BGal4, in spz^2^ vs. 24BGal4>DNT1CK3’+, in spz^2^ | 1283, 237 | 0.261 | ns |
| 2A | Chi-Square, Bonferroni | SPSS 21 | Survival index: 24BGal4, in spz^2^ vs. 24BGal4>DNT2CK, in spz^2^ | 1283, 130 | 0.000 | *** |
| 2B | Chi-Square | SPSS 21 | Survival index: all genotypes |  | 0.000 | *** |
| 2B | Chi-Square, Bonferroni | SPSS 21 | Survival index: DNT1^41^,DNT2^e03444^ vs. elavp35 9.1, in DNT1^41^,DNT2^e03444^ | 504, 915 | 0.000 | *** |
| 2B | Chi-Square, Bonferroni | SPSS 21 | Survival index: chaGal4, in DNT1^41^,DNT2^e03444^ vs. chaGal4>p35, in DNT1^41^,DNT2^e03444^ | 264, 128 | 0.000 | *** |
| 2B | Chi-Square, Bonferroni | SPSS 21 | Survival index: elavGal4, in DNT1^41^,DNT2^e03444^ vs. elavGal4>p35, in DNT1^41^,DNT2^e03444^ | 405, 741 | 0.000 | *** |
| 2B | Chi-Square, Bonferroni | SPSS 21 | Survival index: elavGal4, in DNT1^41^,DNT2^e03444^ vs. elavGal4>DNT1CK3'+, in DNT1^41^,DNT2^e03444^ | 405, 60 | 0.000 | *** |
| 2B | Chi-Square, Bonferroni | SPSS 21 | Survival index: elavGal4, in DNT1^41^,DNT2^e03444^ vs. elavGal4>DNT2CK, in DNT1^41^,DNT2^e03444^ | 405, 245 | 0.000 | *** |
| 2B | Chi-Square, Bonferroni | SPSS 21 | Survival index: elavGal4, in DNT1^41^,DNT2^e03444^ vs. elavGal4>spzCK, in DNT1^41^,DNT2^e03444^ | 405, 1098 | 0.000 | *** |
| 2B | Chi-Square, Bonferroni | SPSS 21 | Survival index: elavGal4, in DNT1^41^,DNT2^e03444^ vs. elavGal4>dTRAF2, in DNT1^41^,DNT2^e03444^ | 405, 1075 | 0.000 | *** |
| 2B | Chi-Square, Bonferroni | SPSS 21 | Survival index: chaGal4, in DNT1^41^,DNT2^e03444^ vs. chaGal4>dTRAF2, in DNT1^41^,DNT2^e03444^ | 264, 215 | 0.000 | *** |
| 2B | Chi-Square, Bonferroni | SPSS 21 | Survival index: 24BGal4, in DNT1^41^,DNT2^e03444^ vs. 24BGal4>DNT2CK, in DNT1^41^,DNT2^e03444^ | 383, 408 | 0.000 | *** |
| 3B | One-Way ANOVA | SPSS 21 | Mean Muscle 4 Boutons/MSA: all genotypes |  | 0.000 | *** |
| 3B | Dunnett | SPSS 21 | Mean Muscle 4 Boutons/MSA: wt vs. spz^2^ | 35, 16 | 0.000 | *** |
| 3B | Dunnett | SPSS 21 | Mean Muscle 4 Boutons/MSA: wt vs. DNT1^41^DNT2^e03444^ | 35, 24 | 1.000 | ns |
| 3B | Dunnett | SPSS 21 | Mean Muscle 4 Boutons/MSA: wt vs. elavGal4>spzCK | 35, 25 | 1.000 | ns |
| 3B | Dunnett | SPSS 21 | Mean Muscle 4 Boutons/MSA: wt vs. elavGal4>DNT1CK3'+ | 35, 26 | 0.990 | ns |
| 3B | Dunnett | SPSS 21 | Mean Muscle 4 Boutons/MSA: wt vs. elavGal4>DNT2CK | 35, 27 | 1.000 | ns |
| 3B | Dunnett | SPSS 21 | Mean Muscle 4 Boutons/MSA: wt vs. 24BGal4>spzCK | 35, 28 | 0.650 | ns |
| 3B | Dunnett | SPSS 21 | Mean Muscle 4 Boutons/MSA: wt vs. 24BGal4>DNT1CK3'+ | 35, 29 | 1.000 | ns |
| 3B | Dunnett | SPSS 21 | Mean Muscle 4 Boutons/MSA: wt vs. 24BGal4>DNT2CK | 35, 30 | 0.966 | ns |
| 3D | One-Way ANOVA | SPSS 21 | Mean Muscle 6,7 Boutons/MSA: all genotypes |  | 0.001 | ** |
| 3D | Dunnett | SPSS 21 | Mean Muscle 6,7 Boutons/MSA: wt vs. spz^2^ | 28, 16 | 0.845 | ns |
| 3D | Dunnett | SPSS 21 | Mean Muscle 6,7 Boutons/MSA: wt vs. DNT1^41^DNT2^e03444^ | 28, 22 | 0.000 | *** |
| 3D | Dunnett | SPSS 21 | Mean Muscle 6,7 Boutons/MSA: wt vs. elavGal4>spzCK | 28, 22 | 0.644 | ns |
| 3D | Dunnett | SPSS 21 | Mean Muscle 6,7 Boutons/MSA: wt vs. elavGal4>DNT1CK3'+ | 28, 20 | 0.098 | ns |
| 3D | Dunnett | SPSS 21 | Mean Muscle 6,7 Boutons/MSA: wt vs. elavGal4>DNT2CK | 28, 20 | 0.329 | ns |
| 3D | Dunnett | SPSS 21 | Mean Muscle 6,7 Boutons/MSA: wt vs. 24BGal4>spzCK | 28, 23 | 0.393 | ns |
| 3D | Dunnett | SPSS 21 | Mean Muscle 6,7 Boutons/MSA: wt vs. 24BGal4>DNT1CK3'+ | 28, 23 | 0.903 | ns |
| 3D | Dunnett | SPSS 21 | Mean Muscle 6,7 Boutons/MSA: wt vs. 24BGal4>DNT2CK | 28, 12 | 0.969 | ns |
| 3E | One-Way ANOVA | SPSS 21 | Axonal terminal length Muscle 4/MSA: all genotypes |  | 0.029 | * |
| 3E | Dunnett | SPSS 21 | Axonal terminal length Muscle 4/MSA: wt vs. spz^2^ | 64, 28 | 0.526 | ns |
| 3E | Dunnett | SPSS 21 | Axonal terminal length Muscle 4/MSA: wt vs. DNT1^41^DNT2^e03444^ | 64, 47 | 0.001 | ** |
| 3E | Dunnett | SPSS 21 | Axonal terminal length Muscle 4/MSA: wt vs. elavGal4>spzCK | 64, 21 | 1.000 | ns |
| 3E | Dunnett | SPSS 21 | Axonal terminal length Muscle 4/MSA: wt vs. elavGal4>DNT1CK3'+ | 64, 20 | 0.996 | ns |
| 3E | Dunnett | SPSS 21 | Axonal terminal length Muscle 4/MSA: wt vs. elavGal4>DNT2CK | 64, 20 | 1.000 | ns |
| 3E | Dunnett | SPSS 21 | Axonal terminal length Muscle 4/MSA: wt vs. 24BGal4>spzCK | 64, 24 | 0.879 | ns |
| 3E | Dunnett | SPSS 21 | Axonal terminal length Muscle 4/MSA: wt vs. 24BGal4>DNT1CK3'+ | 64, 22 | 0.810 | ns |
| 3E | Dunnett | SPSS 21 | Axonal terminal length Muscle 4/MSA: wt vs. 24BGal4>DNT2CK | 64, 12 | 0.896 | ns |
| 3G | One-Way ANOVA | SPSS 21 | Axonal terminal length Muscle 6,7/MSA: all genotypes |  | 0.000 | *** |
| 3G | Dunnett | SPSS 21 | Axonal terminal length Muscle 6,7/MSA: wt vs. spz^2^ | 53, 16 | 0.130 | ns |
| 3G | Dunnett | SPSS 21 | Axonal terminal length Muscle 6,7/MSA: wt vs. DNT1^41^DNT2^e03444^ | 53, 45 | 0.000 | *** |
| 3G | Dunnett | SPSS 21 | Axonal terminal length Muscle 6,7/MSA: wt vs. elavGal4>spzCK | 53, 22 | 0.000 | *** |
| 3G | Dunnett | SPSS 21 | Axonal terminal length Muscle 6,7/MSA: wt vs. elavGal4>DNT1CK3'+ | 53, 20 | 0.000 | *** |
| 3G | Dunnett | SPSS 21 | Axonal terminal length Muscle 6,7/MSA: wt vs. elavGal4>DNT2CK | 53, 20 | 0.001 | ** |
| 3G | Dunnett | SPSS 21 | Axonal terminal length Muscle 6,7/MSA: wt vs. 24BGal4>spzCK | 53, 22 | 0.356 | ns |
| 3G | Dunnett | SPSS 21 | Axonal terminal length Muscle 6,7/MSA: wt vs. 24BGal4>DNT1CK3'+ | 53, 23 | 0.210 | ns |
| 3G | Dunnett | SPSS 21 | Axonal terminal length Muscle 6,7/MSA: wt vs. 24BGal4>DNT2CK | 53, 12 | 0.128 | ns |
| 4C | One-Way ANOVA | SPSS 21 | Mean Muscle 4 Active zones per bouton: all genotypes |  | 0.000 | *** |
| 4C | Dunnett | SPSS 21 | Mean Muscle 4 Active zones per bouton: wt vs. spz^2^ | 19, 8 | 0.000 | *** |
| 4C | Dunnett | SPSS 21 | Mean Muscle 4 Active zones per bouton: wt vs. DNT1^41^,DNT2^e03444^ | 19, 23 | 0.001 | ** |
| 4C | One-Way ANOVA | SPSS 21 | Mean Muscle 6,7 Active zones per bouton: all genotypes |  | 0.000 | *** |
| 4C | Dunnett | SPSS 21 | Mean Muscle 6,7 Active zones per bouton: wt vs. spz^2^ | 19, 8 | 0.256 | ns |
| 4C | Dunnett | SPSS 21 | Mean Muscle 6,7 Active zones per bouton: wt vs. DNT1^41^,DNT2^e03444^ | 19, 23 | 0.000 | *** |
| 4D | One-Way ANOVA | SPSS 21 | Mean Muscle 4 Active zones/terminal length: all genotypes |  | 0.023 | * |
| 4D | Dunnett | SPSS 21 | Mean Muscle 4 Active zones/terminal length: wt vs. spz^2^ | 19, 8 | 0.046 | * |
| 4D | Dunnett | SPSS 21 | Mean Muscle 4 Active zones/terminal length: wt vs. DNT1^41^,DNT2^e03444^ | 19, 23 | 0.033 | * |
| 4D | One-Way ANOVA | SPSS 21 | Mean Muscle 6,7 Active zones/terminal length: all genotypes |  | 0.000 | *** |
| 4D | Dunnett | SPSS 21 | Mean Muscle 6,7 Active zones/terminal length: wt vs. spz^2^ | 19, 8 | 0.061 | ns |
| 4D | Dunnett | SPSS 21 | Mean Muscle 6,7 Active zones/terminal length: wt vs. DNT1^41^,DNT2^e03444^ | 19, 23 | 0.000 | *** |
| 4E | One-Way ANOVA | SPSS 21 | DeadEasy Synapse voxel mean Muscle 4: all genotypes |  | 0.006 | ** |
| 4E | Dunnett | SPSS 21 | DeadEasy Synapse voxel mean Muscle 4: wt vs. spz^2^ | 19, 8 | 0.003 | ** |
| 4E | Dunnett | SPSS 21 | DeadEasy Synapse voxel mean Muscle 4: wt vs. DNT1^41^DNT2^e03444^ | 19, 23 | 0.634 | ns |
| 4E | One-Way ANOVA | SPSS 21 | DeadEasy Synapse voxel mean Muscle 6,7: all genotypes |  | 0.009 | ** |
| 4E | Dunnett | SPSS 21 | DeadEasy Synapse voxel mean Muscle 6,7: wt vs. spz^2^ | 19, 8 | 0.168 | ns |
| 4E | Dunnett | SPSS 21 | DeadEasy Synapse voxel mean Muscle 6,7: wt vs. DNT1^41^DNT2^e03444^ | 19, 23 | 0.005 | ** |
| 4F | One-Way ANOVA | SPSS 21 | DeadEasy Synapse voxel/terminal length Muscle 4: all genotypes |  | 0.061 | ns |
| 4F | Dunnett | SPSS 21 | DeadEasy Synapse voxel/terminal length Muscle 4: wt vs. spz^2^ | 19, 8 | 0.053 | ns |
| 4F | Dunnett | SPSS 21 | DeadEasy Synapse voxel/terminal length Muscle 4: wt vs. DNT1^41^DNT2^e03444^ | 19, 23 | 0.161 | ns |
| 4F | One-Way ANOVA | SPSS 21 | DeadEasy Synapse voxel/terminal length Muscle 6,7: all genotypes |  | 0.000 | *** |
| 4F | Dunnett | SPSS 21 | DeadEasy Synapse voxel/terminal length Muscle 6,7: wt vs. spz^2^ | 19, 8 | 0.509 | ns |
| 4F | Dunnett | SPSS 21 | DeadEasy Synapse voxel/terminal length Muscle 6,7: wt vs. DNT1^41^DNT2^e03444^ | 19, 23 | 0.000 | *** |
| 4G | One-Way ANOVA | SPSS 21 | DeadEasy Synapse voxel mean Muscle 6,7: all genotypes |  | 0.001 | ** |
| 4G | Dunnett | SPSS 21 | DeadEasy Synapse voxel mean Muscle 6,7: wt vs. DNT1^41^,Df(3L)6092/DNT1^55^,DNT2^e03444^ | 12, 16 | 0.001 | ** |
| 4G | Dunnett | SPSS 21 | DeadEasy Synapse voxel mean Muscle 6,7: wt vs. elavGal4> DNT1CK3'+, per bouton in DNT1^41^,DNT2^e03444^ | 12, 18 | 0.446 | ns |
| 4G | Dunnett | SPSS 21 | DeadEasy Synapse voxel mean Muscle 6,7: wt vs. elavGal4> DNT2CK, per bouton in DNT1^41^,DNT2^e03444^ | 12, 10 | 0.994 | ns |
| 4G | One-Way ANOVA | SPSS 21 | Mean Muscle 6,7 Active zones per bouton: all genotypes |  | 0.000 | *** |
| 4G | Dunnett | SPSS 21 | Mean Muscle 6,7 Active zones per bouton: wt vs. DNT1^41^,Df(3L(6092)/DNT1^55^,DNT2^e03444^ | 12, 16 | 0.009 | ** |
| 4G | Dunnett | SPSS 21 | Mean Muscle 6,7 Active zones per bouton: wt vs. DNT1^41^,DNT2^e03444^,elavGal4xUASDNT1CK3'+ | 12, 18 | 0.074 | ns |
| 4G | Dunnett | SPSS 21 | Mean Muscle 6,7 Active zones per bouton: wt vs. DNT1^41^,DNT2^e03444^,elavGal4xUASDNT2CK6a | 12, 10 | 0.002 | ** |
| 4H | One-Way ANOVA | SPSS 21 | DeadEasy Synapse voxel/terminal length Muscle 6,7: all genotypes |  | 0.000 | *** |
| 4H | Dunnett | SPSS 21 | DeadEasy Synapse voxel/terminal length Muscle 6,7: wt vs. DNT1^41^,Df(3L)6092/DNT155,DNT2^e03444^ | 12, 16 | 0.001 | ** |
| 4H | Dunnett | SPSS 21 | DeadEasy Synapse voxel/terminal length Muscle 6,7: wt vs. elavGal4> DNT1CK3'+, in DNT1^41^,DNT2^e03444^ | 12, 18 | 0.479 | ns |
| 4H | Dunnett | SPSS 21 | DeadEasy Synapse voxel/terminal length Muscle 6,7: wt vs. elavGal4> DNT2CK, in DNT1^41^,DNT2^e03444^ | 12, 10 | 0.995 | ns |
| 4H | One-Way ANOVA | SPSS 21 | Mean Muscle 6,7 Active zones/terminal length: all genotypes |  | 0.015 | * |
| 4H | Dunnett | SPSS 21 | Mean Muscle 6,7 Active zones/terminal length: wt vs. DNT1^41^,Df(3L(6092)/DNT155,DNT2^e03444^ | 12, 16 | 0.036 | * |
| 4H | Dunnett | SPSS 21 | Mean Muscle 6,7 Active zones/terminal length: wt vs. DNT1^41^,DNT2^e03444^,elavGal4xUASDNT1CK3'+ | 12, 18 | 1.000 | ns |
| 4H | Dunnett | SPSS 21 | Mean Muscle 6,7 Active zones/terminal length: wt vs. DNT1^41^,DNT2^e03444^,elavGal4xUASDNT2CK6a | 12, 10 | 0.988 | ns |
| 5C | Chi-Square | SPSS 21 | % Muscle 6,7 NMJ with debris: all genotypes |  | 0.001 | ** |
| 5C | Chi-Square, Bonferroni | SPSS 21 | % Muscle 6,7 NMJ with debris: wt vs. spz^2^ | 28, 16 | 0.30 | ns |
| 5C | Chi-Square, Bonferroni | SPSS 21 | % Muscle 6,7 NMJ with debris: wt vs. DNT1^41^DNT2^e03444^ | 28, 22 | 0.035 | * |
| 5C | Chi-Square, Bonferroni | SPSS 21 | % Muscle 6,7 NMJ with debris: wt vs. elavGal4>spzCK | 28, 21 | 0.000 | *** |
| 5C | Chi-Square, Bonferroni | SPSS 21 | % Muscle 6,7 NMJ with debris: wt vs. elavGal4>DNT1CK3'+ | 28, 20 | 0.785 | ns |
| 5C | Chi-Square, Bonferroni | SPSS 21 | % Muscle 6,7 NMJ with debris: wt vs. elavGal4>DNT2CK | 28, 20 | 0.07 | ns |
| 5D | Chi-Square | SPSS 21 | % Muscle 6,7 NMJ with ≥1 ghost boutons: all genotypes |  | 0.001 | ** |
| 5D | Chi-Square, Bonferroni | SPSS 21 | % Muscle 6,7 NMJ with ≥1 ghost boutons: wt vs. spz^2^ | 28, 16 | 2.345 | ns |
| 5D | Chi-Square, Bonferroni | SPSS 21 | % Muscle 6,7 NMJ with ≥1 ghost boutons: wt vs. DNT1^41^DNT2^e03444^ | 28, 31 | 0.030 | * |
| 5D | Chi-Square, Bonferroni | SPSS 21 | % Muscle 6,7 NMJ with ≥1 ghost boutons: wt vs. elavGal4>spzCK | 28, 22 | 0.605 | ns |
| 5D | Chi-Square, Bonferroni | SPSS 21 | % Muscle 6,7 NMJ with ≥1 ghost boutons: wt vs. elavGal4>DNT1CK3'+ | 28, 20 | 0.01 | * |
| 5D | Chi-Square, Bonferroni | SPSS 21 | % Muscle 6,7 NMJ with ≥1 ghost boutons: wt vs. elavGal4>DNT2CK | 28, 20 | 3.18 | ns |
| 6C | Students t-test | Graphpad Prism 6 | Muscle 4 mEJP frequency 1.8mM Ca^2+^: wt vs. spz^2^ | 8, 8 | 0.672 | ns |
| 6C | Students t-test | Graphpad Prism 6 | Muscle 4 mEJP frequency 0.7mM Ca^2+^: wt vs. spz^2^ | 8, 8 | 0.003 | ** |
| 6C | Students t-test | Graphpad Prism 6 | Muscle 4 mEJP amplitude 1.8mM Ca^2+^: wt vs. spz^2^ | 8, 8 | 0.152 | ns |
| 6C | Students t-test | Graphpad Prism 6 | Muscle 4 mEJP amplitude 0.7mM Ca^2+^: wt vs. spz^2^ | 8, 8 | 0.010 | * |
| 6D | Students t-test | Graphpad Prism 6 | Muscle 4 EJP amplitude 1.8mM Ca^2+^: wt vs. spz^2^ | 8, 8 | 0.379 | ns |
| 6D | Students t-test | Graphpad Prism 6 | Muscle 4 EJP amplitude 0.7mM Ca^2+^: wt vs. spz^2^ | 8, 8 | 0.098 | ns |
| 6D | Students t-test | Graphpad Prism 6 | Muscle 4 Quantal content 0.7mM Ca^2+^: wt vs. spz^2^ | 8, 8 | 0.422 | ns |
| 6G | Students t-test | Graphpad Prism 6 | Muscle 6,7 mEJP frequency 1.8mM Ca2+: wt vs. DNT1^55^,DNT2^e03444^ | 14, 9 | 0.073 | ns |
| 6G | Students t-test | Graphpad Prism 6 | Muscle 6,7 mEJP frequency 0.7mM Ca2+: wt vs. DNT1^55^,DNT2^e03444^ | 9, 10 | 0.244 | ns |
| 6G | Students t-test | Graphpad Prism 6 | Muscle 6,7 mEJP amplitude 1.8mM Ca2+: wt vs. DNT1^55^,DNT2^e03444^ | 13, 10 | 0.332 | ns |
| 6G | Students t-test | Graphpad Prism 6 | Muscle 6,7 mEJP amplitude 0.7mM Ca2+: wt vs. DNT1^55^,DNT2^e03444^ | 9, 10 | 0.293 | ns |
| 6F | Students t-test | Graphpad Prism 6 | Muscle 6,7 EJP amplitude 1.8mM Ca2+: wt vs. DNT1^55^,DNT2^e03444^ | 9, 10 | 0.543 | ns |
| 6F | Students t-test | Graphpad Prism 6 | Muscle 6,7 EJP amplitude 0.7mM Ca2+: wt vs. DNT1^55^,DNT2^e03444^ | 9, 10 | 0.119 | ns |
